# Non-linear relationships in clinical research

**DOI:** 10.1093/ndt/gfae187

**Published:** 2024-08-21

**Authors:** Nicholas C Chesnaye, Merel van Diepen, Friedo Dekker, Carmine Zoccali, Kitty J Jager, Vianda S Stel

**Affiliations:** ERA Registry, Amsterdam UMC location University of Amsterdam, Medical Informatics, Amsterdam, The Netherlands; Amsterdam Public Health Research Institute, Quality of Care, Amsterdam, The Netherlands; Department of Clinical Epidemiology, Leiden University Medical Center, Leiden, The Netherlands; Department of Clinical Epidemiology, Leiden University Medical Center, Leiden, The Netherlands; Associazione Ipertensione Nefrologia Trapianto Renale (IPNET), c/o Nefrologia, Grande Ospedale Metropolitano, Reggio Calabria, Italy; ERA Registry, Amsterdam UMC location University of Amsterdam, Medical Informatics, Amsterdam, The Netherlands; Amsterdam Public Health Research Institute, Quality of Care, Amsterdam, The Netherlands; ERA Registry, Amsterdam UMC location University of Amsterdam, Medical Informatics, Amsterdam, The Netherlands; Amsterdam Public Health Research Institute, Quality of Care, Amsterdam, The Netherlands

**Keywords:** dichotomania, generalized additive models, non-linear relationship, polynomials, splines, transformations

## Abstract

True linear relationships are rare in clinical data. Despite this, linearity is often assumed during analyses, leading to potentially biased estimates and inaccurate conclusions. In this introductory paper, we aim to first describe—in a non-mathematical manner—how to identify non-linear relationships. Various methods are then discussed that can be applied to deal with non-linearity, including transformations, polynomials, splines and generalized additive models, along with their strengths and weaknesses. Finally, we illustrate the use of these methods with a practical example from nephrology, providing guidance on how to report the results from non-linear relationships.

## INTRODUCTION

In clinical research, we often aim to assess the strength of relationships between a variable of interest (i.e. the independent variable, for example blood pressure) and an outcome variable (i.e. the dependent variable, for example mortality). When dealing with a continuous independent variable, a linear relationship with the outcome is often assumed, mostly due to the ease of understanding, interpretation and communication [1]. A linear relationship implies that a change in the continuous independent variable is directly proportional to the change in the outcome, and will therefore create a straight (i.e. linear) line when drawn. True linear relationships do exist (e.g. between time and distance when moving at a constant speed), but rarely in biology; complex systems, interactions, threshold effects, feedback mechanisms and biological variation may all give way to non-linear relationships [1, 2]. In nephrology, these types of relationships are also ubiquitous. For one, longitudinal trajectories over time (e.g. for various lab values, or renal decline) are almost exclusively non-linear, especially on the individual level. Relationships between risk factors and adverse outcomes are also often non-linear. For instance, the relationship between hemoglobin A1c (HbA1c) and mortality risk is J-shaped, meaning that patients with high HbA1c levels have the highest mortality risk, while patients with low HbA1c are also exposed to a high risk, albeit less than that associated with high HbA1c [3]. The association between serum potassium and mortality risk is U-shaped [4], as is the association between body mass index (BMI) and adverse outcomes [5], meaning that the highest risk is found at both extremes of the risk factor, with a zone of lowest risk in the middle. Nonetheless, despite numerous examples of non-linear relationships occurring in clinical data, linearity is still assumed more often than not. However, if the true underlying relationship is non-linear, this simplification can lead to a loss of statistical power, biased estimates and inaccurate conclusions. The consequences of incorrectly assuming a linear relationship may differ depending on the type of study. In clinical epidemiology, a broad distinction is made between causal (i.e. etiological) studies and studies focused on prediction [6]. For instance, incorrectly assuming a linear relationship between a continuous predictor and the outcome in a clinical prediction model could reduce predictive accuracy [7]. In causal research, assuming linearity in non-linear confounder–outcome or confounder–exposure relationships can lead to residual confounding [8]. In this introductory paper, we describe—in a non-mathematical manner—how to identify non-linear relationships, and the various methods that can be applied to deal with non-linearity. We discuss the strengths and weaknesses of each method, and give an example from nephrology on how to apply these methods.

### Identifying non-linear relationships

**Figure 1:**
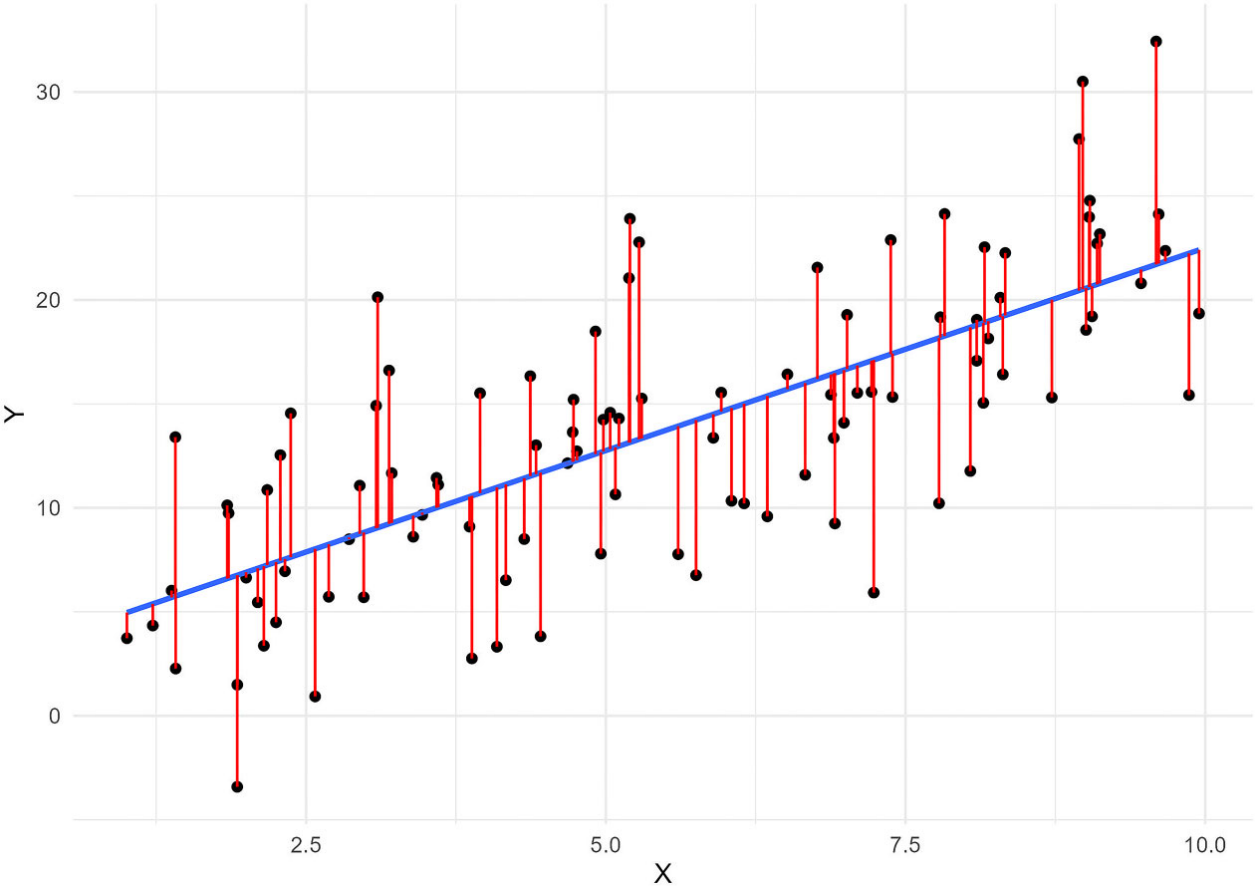
A linear regression model fits a straight line (in blue) as close as possible to every data point (in black) on the scatterplot. The difference between each data point and the line produced by the model is called the “residual,” represented here by the red lines.

**Figure 2:**
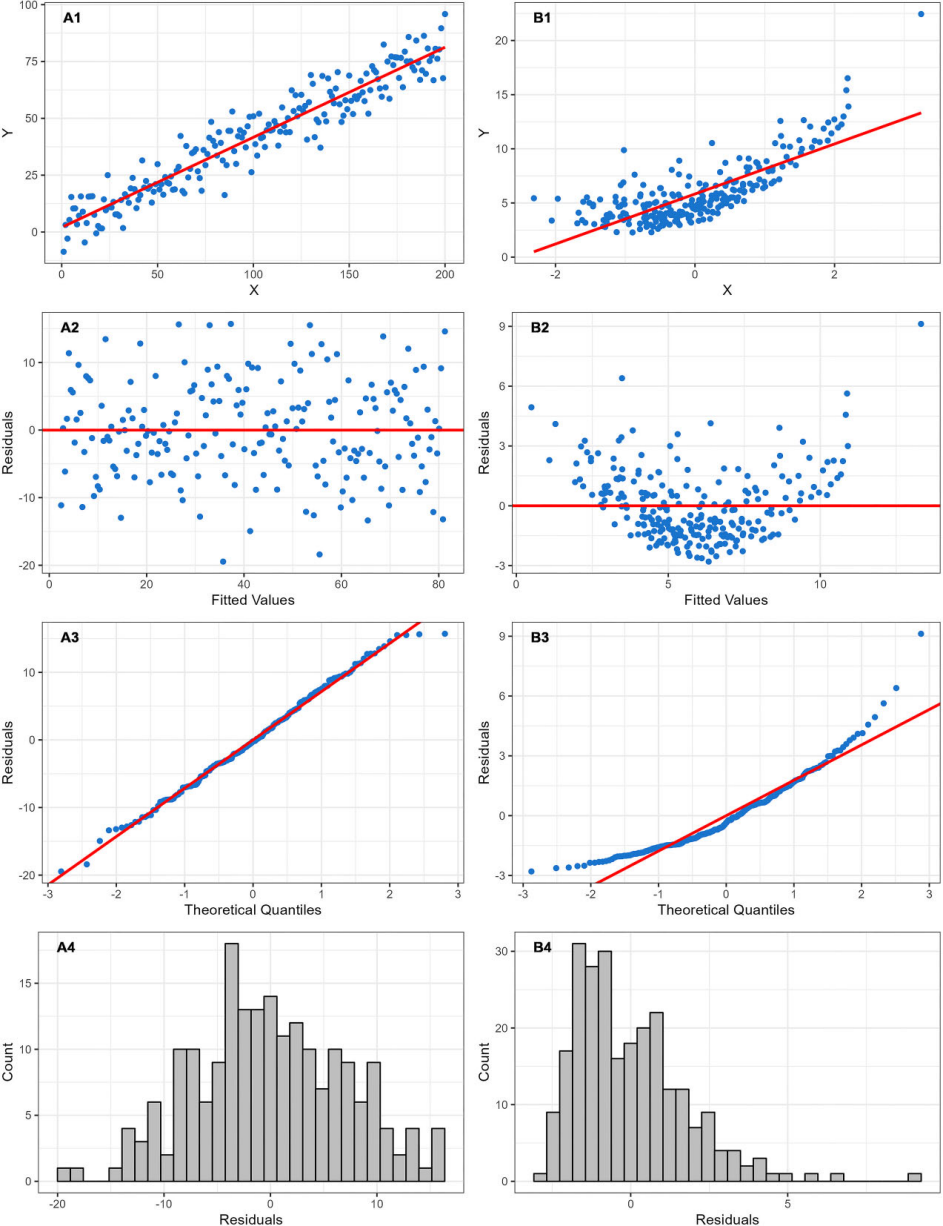
Scatterplots with linear regression line, residual plots, normal probability plots and histograms of residuals for both an example of a linear relationship, and an example of a non-linear relationship in which linear regression clearly does not fit the data. In the linear example (**A**), the residuals are spread randomly around the regression line (A1–A3), following an approximate normal distribution (A4), indicating a constant variance of residuals (i.e. homoscedasticity). In the non-linear example (**B**), a pattern is visible in the distribution of residuals (B1–B3), and the residuals follow a skewed, non-normal, distribution (B4), indicating heteroscedasticity—an important departure from the linear regression assumption of constant variance of residuals.

An important first step during data analysis is to assess patterns in the data using visual methods. A scatterplot of the independent continuous variable on the X-axis and the continuous outcome on the Y-axis may help reveal any non-linear patterns in the data. To further examine potential non-linearity, a linear regression model can be used to fit a straight line as close as possible to every data point on the scatterplot. Deviations from this line can indicate non-linearity. These deviations, known as “residuals,” are the differences between each data point and the line produced by the model (Fig. 1). A residual plot is a scatterplot of the residuals versus the regression line, and is often used to assess linearity, identify influential data points (i.e. outliers) and check other model assumptions. Ideally, residuals should be randomly distributed across the plot around the regression line (following an approximate normal distribution), indicating a constant variance of residuals (i.e. homoscedasticity). Any visible patterns in residuals are indicative of an unequal variance in residuals across the range of the independent variable (i.e. heteroscedasticity), and require further investigation. This can also be visually assessed using a “normal probability plot” of residuals which plots the residuals against the expected value of the residual when it would follow a normal distribution. Of note, it is important to use the full model containing all relevant covariates when using residuals to assess the form of the relationship of interest. Figure 2 provides scatterplots, residual plots, normal probability plots and a histogram of the distribution of residuals, for both an example of a linear relationship with constant variance in the residuals, and an example of a non-linear relationship in which a linear regression line clearly does not fit the data. These plots can be reproduced using the R code provided in the Supplementary data. In addition, the methods we describe below (e.g. splines) to address non-linearity during analysis can also be used exploratively to visualize relationships and help reveal non-linear patterns [9].

It should be noted that linear regression is not the only model that relies on a linear relationship; this assumption is common across many statistical models. In the case of linear regression, the model coefficient is interpreted as the change in outcome for a one-unit increase in the independent variable, and that this effect is constant across the full range of the independent variable. Logistic regression, for instance, used to model binary outcomes, also relies on the assumption of a linear relationship between a continuous independent variable and the log-odds—or logit—of the outcome occurring [10]. Similar to linear regression, the modeled coefficient is interpreted as the change in the log-odds of the outcome occurring for every unit increase in the continuous independent variable. A residual plot of the continuous independent variable on the X-axis and the residuals on the Y-axis may help reveal any non-linear patterns in logistic regression. As mentioned above, splines can also be used exploratively to visualize the relationship between the continuous independent variable on the X-axis and the log odds on the Y-axis.

## METHODS FOR ADDRESSING NON-LINEAR RELATIONSHIPS

### Dichotomization and categorization

A straightforward approach to handle non-linearity between a continuous independent variable and the outcome is to categorize the independent variable based on meaningful cut-off values, allowing the outcome to vary over these categories. Cut-off values can be selected by using established guidelines, such as classifying renal function measured by estimated glomerular filtration rate (eGFR) as chronic kidney disease (CKD) when below 60 mL/min/1.73 m^2^ and as non-CKD when above this threshold [11]. In the absence of clinically motivated cut-off points, quartiles or quintiles are often used [12], or the median if two groups are of interest. The main advantage of categorization is to simplify a non-linear relationship by transforming the continuous independent variable into easier to understand groups. This can help improve communication of results to patients, policy makers and other stakeholders. For this reason, categorization is often used for risk classification.

**Figure 3:**
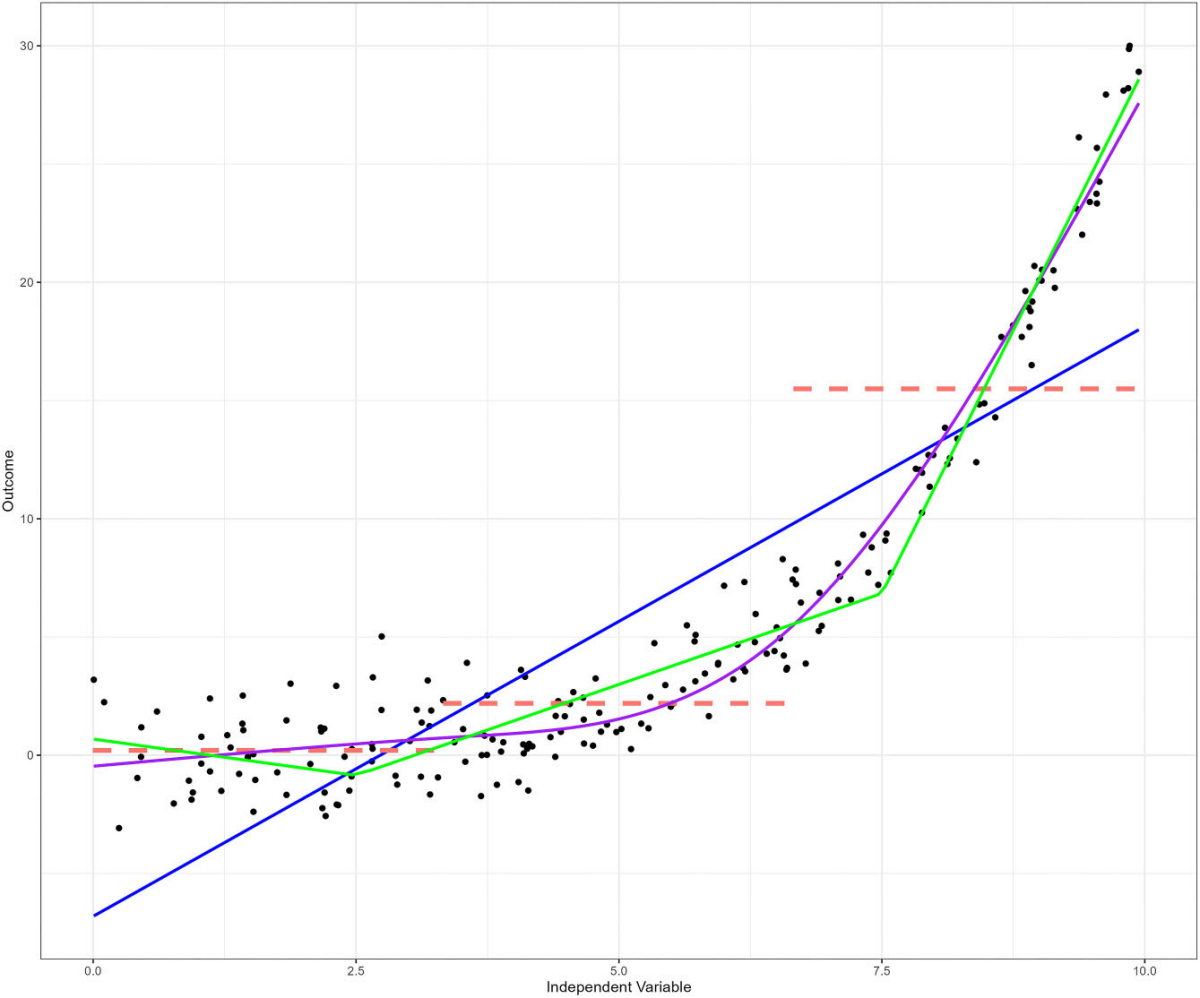
The raw data points (in black) clearly indicate a non-linear relationship between the independent variable and the outcome, which is poorly fit by a linear regression line (in blue). Categorization according to tertiles of the independent variable (in red) seems to better reflect the relationship between the two variables, particularly for the lower two tertiles. However, the third tertile does not reflect the underlying data very well, and may obscure the underlying trend as it encompasses a wide range of outcome values. A simple linear spline with knots placed at *x* = 2.5 and *x* = 7.5 provides more flexibility, and a better representation of the data (green). A restricted cubic spline function fits the data best (purple), with knots placed at tertiles of the independent variable.

Many have warned against the dangers of categorization and “dichotomania”—the compulsion to inappropriately dichotomize continuous data—[13] in clinical research [14–17]. While categorization offers simplicity and interpretability, it comes at the cost of information loss [12, 17]. Continuous variables contain more detailed information, as they can take on any value within a range, whereas categorization risks treating all outcomes within a category as identical, potentially hiding important variations in the outcome within each category. Figure 3 illustrates that categorizing the independent variable into tertiles better reflects the underlying non-linear relationship than a linear model, especially within the lower two tertiles. However, the third tertile obscures the underlying sharp upward trend as it encompasses a wide range of outcome values. This plot can be reproduced using the R code provided in the Supplementary data.

The consequences of categorization differ depending on the type of study [6]. In prediction models, categorization of an important predictor may mask valuable predictive information, limiting model accuracy [15–18]. Increasing the number of categories may help recover part of this information, but at the loss of statistical power. Similarly, in causal research, adjusting for a continuous confounder using categorization can lead to residual confounding due to the loss of information caused by categorization. An additional issue arises with changes in the independent variable at the threshold of each category, which may lead to abrupt, stepwise changes in the outcome. For example, age-adapted CKD definitions use eGFR thresholds to define CKD by age group [19]. While the advantage of these definitions is that they account for normal age-related decline in kidney function, they can lead to abrupt, unrealistic, changes in CKD diagnosis status around birthdays, as a result of which they move from one age category to another, even without a change in their eGFR, which is problematic.

### Transformations

**Figure 4:**
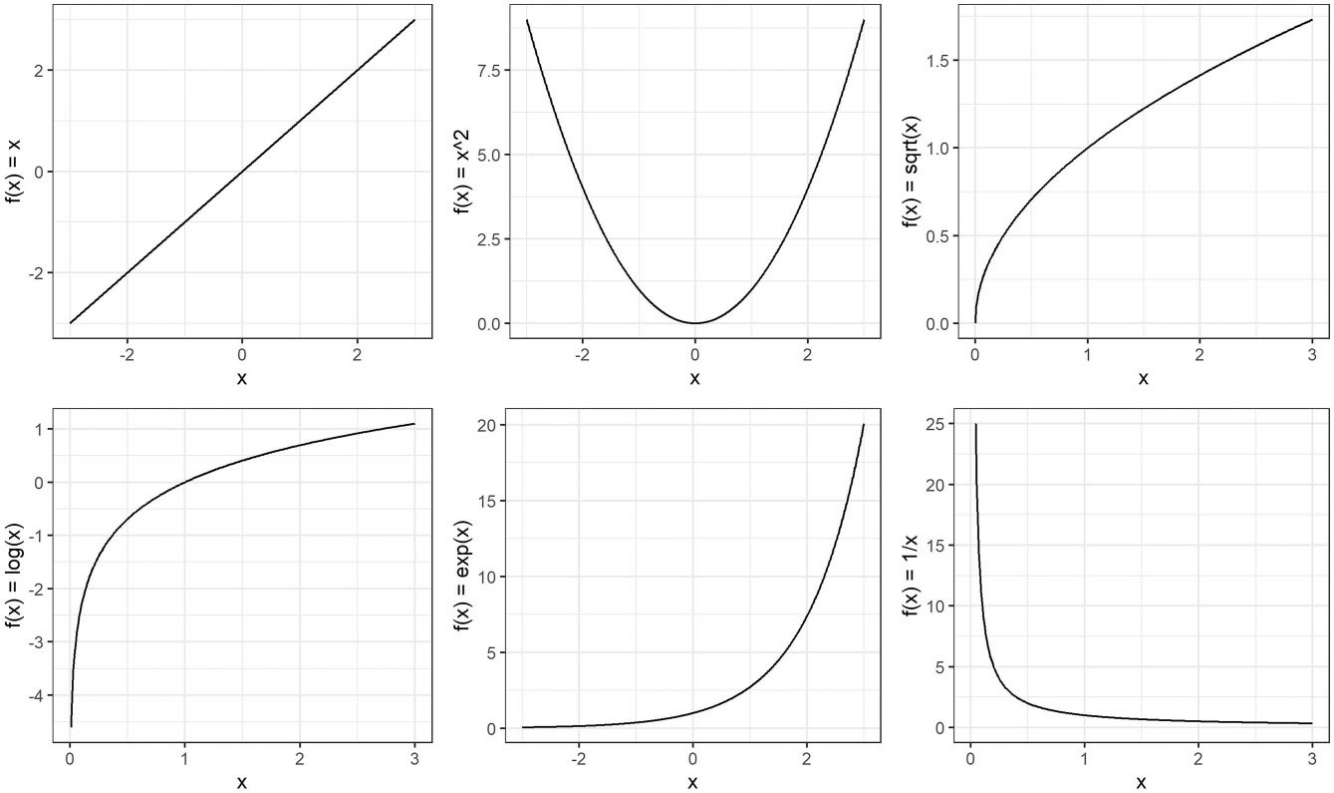
Changing the distribution of a variable by applying some mathematical function to each value. Common transformations of *x* are illustrated by changing the distribution using the logarithm (log *x*), square (*x*^2^), reciprocal (1/*x*), square root (√*x*) and exponential transformations (exp *x*).

Transformation is another simple yet effective approach to addressing non-linearity, especially if the shape of the relationship is known. Transformation is a technique used to change the distribution of a variable by applying some mathematical function. By modifying the distribution—essentially creating a new version of the variable—transformation can help create a (more) linear relationship. Of note, transformations can be applied not only to the independent variable, but also to the outcome (in cases where the outcome is continuous). Transformations are also used to deal with other departures from model assumptions such as heteroscedasticity. Transformations are especially useful when the data follows a certain (known) distribution. While they cannot handle every possible shape, transformations offer a flexible way to achieve linearity for many common cases. Common transformations include the logarithm, square, reciprocal, square root and exponential transformations. As mentioned previously, scatter and residual plots can help identify patterns in the data and help choose the right transformation. Figure 4 illustrates the effect of common transformations that can be applied to help linearize a non-linear relationship. In practice, it helps to experiment with different transformations, and evaluate scatter plots, residual plots and model fit metrics (as described in the example below) to help choose the right one. It is important to keep in mind that transformations change the interpretation of the model coefficients. For instance, the coefficient of a log-transformed independent variable no longer corresponds with a simple linear change, but now translates to a percentage change in the outcome. The UCLA website provides clear guidance on how to interpret model coefficients in cases where either the independent or the dependent (or both) are log-transformed [20].

### Polynomial functions

In Fig. 4, we provide an example of a simple power transformation using the square function, which raises a variable to the power of 2 (*x*^2^), providing a U-shaped curve. Higher powers of *x* can be also used to determine the shape of the function, such as the cubic function (*x*^3^) which has a distinctive S-shape. These power terms form the core elements of so-called polynomial functions, which along with the coefficients included in the function, offer a flexible way to model various curves. By adjusting the coefficients and power functions, polynomials offer a wide variety of shapes. “Fractional” polynomial functions allow the power terms to be fractions instead of just whole numbers (i.e. *x*^1/2^) [21]. A (fractional) polynomial function often provides sufficient flexibility to follow relatively simple non-linear curves, providing a simpler solution than the more advanced techniques we describe below. However, higher degree polynomials can be sensitive to “noise” in the data, and are not suited for fitting some of the more complex curves (e.g. sudden shifts, discontinuities, or logarithmic curves) [21]. They may also suffer from Runge's phenomenon, becoming unstable and oscillating at the edges of the data, and extrapolate poorly beyond the original range of the independent variable [22].

### Regression splines

A powerful approach to dealing with non-linearity is provided by the family of spline functions. Instead of a single function defining the whole curve, such as polynomials or other transformations, splines are constructed by using a series of functions, each defining a different segment of the curve. As splines are constructed segment by segment—or piece by piece—they are often referred to as “piecewise” functions. Segments are connected to each other using so-called “knots,” and the spline is restricted to join at these knots so there are no gaps in the curve [14]. The simplest spline function is the linear spline function. This function assumes linearity within each segment, but the overall curve formed by the connected segments can be non-linear [23]. For a small number of segments, linear spline models can be as easy to interpret as linear regression, as each segment can be represented by a single slope. Figure 3 provides an example of a simple linear spline with two knots (in green). These two knots divide the range of the independent variable into three segments, each with its own slope.

**Figure 5:**
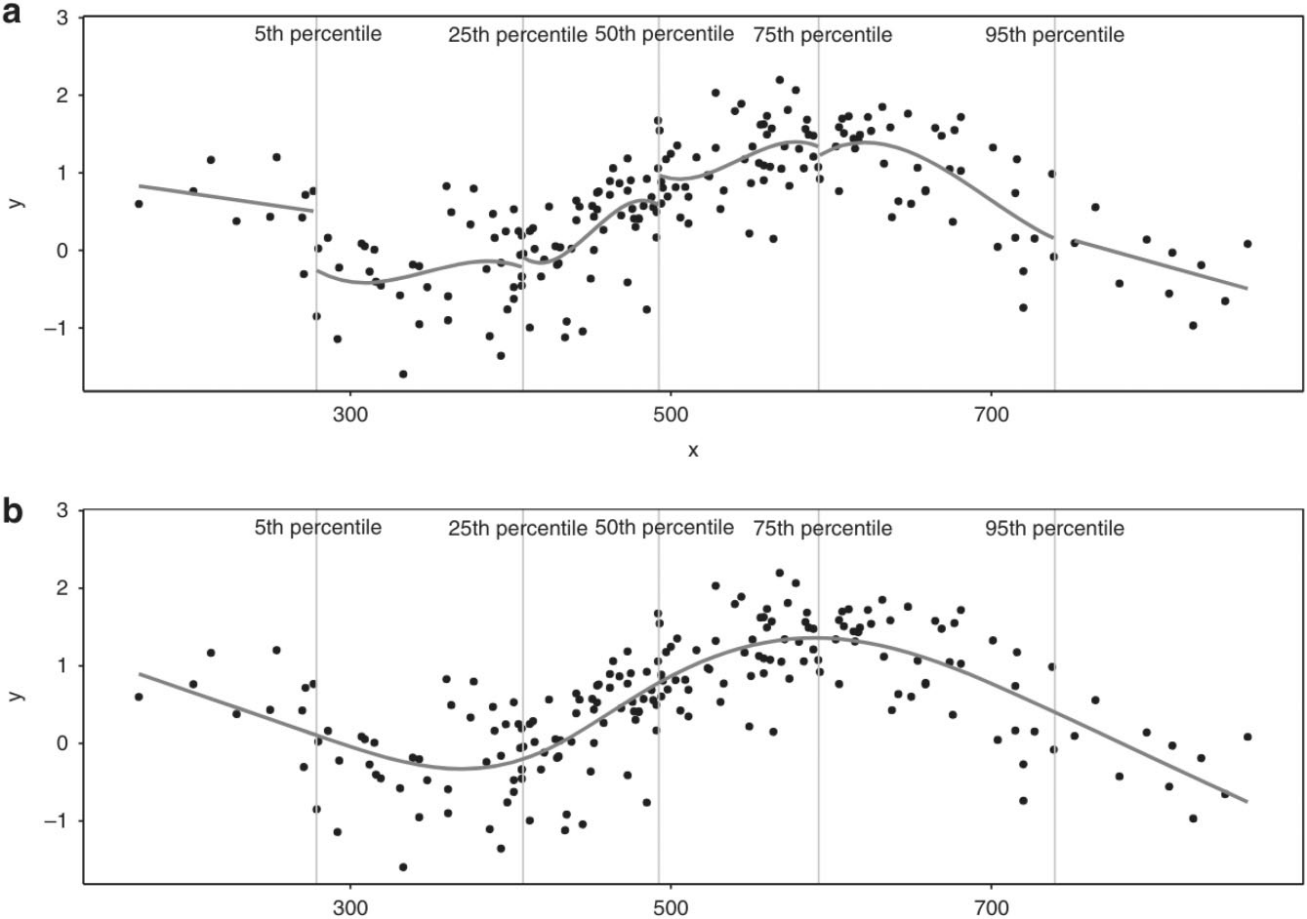
A restricted cubic spline. Panel (**A**) illustrates how a cubic spline is constructed segment by segment, with the X-axis segmented by the 5th, 25th, 50th, 75th and 95th percentiles, using cubic polynomials to define the shape of each segment. As the cubic spline is “restricted,” the first (before the 5th percentile) and last (after the 95th percentile) segment are forced to be linear. Panel (**B**) shows how segments are connected to each other using knots (placed at the 5th, 25th, 50th, 75th and 95th percentiles), and shows how the spline is restricted to join at these knots so there are no gaps in the curve. Figure copied from: Gauthier J, Wu, QV, Gooley TA. Cubic splines to model relationships between continuous variables and outcomes: a guide for clinicians. *Bone Marrow Transplant* 55, 675–680 (2020). https://doi.org/10.1038/s41409-019-0679-x.

A more flexible and smooth type of spline is the “cubic spline,” which includes cubic polynomials to define the shape for each segment (Fig. 5). As this type of spline is known to behave erratically before the first knot and after the last knot (i.e. in the “tails”), the spline function is commonly forced to be linear in these segments and is then referred to as a restricted cubic spline or a natural spline (also presented in Fig. 3 in purple) [24]. The number of knots, and their placement, are important for defining the shape and flexibility of the spline, with more knots placed closer together allowing for sharper changes in curvature. It is common practice to place knots at quantiles of the independent variable, but knot location may also be determined based on theoretical or clinical relevance [14]. For example, de Rooij *et al*. studied quality of life trajectories before and after dialysis initiation in advanced CKD patients [25]. To capture the hypothesized non-linear change in quality of life surrounding the start of dialysis, the authors used a three-knot restricted cubic spline function with knots placed at the start of dialysis, a half-year before and a half-year after [25]. Although placement is important, the number of knots has more impact on model fit than the exact knot locations [24]. Deciding on the number of knots is a trade-off; increasing the number of knots allows the spline to follow more complex patterns in the data, but this comes at the cost of increased risk of overfitting. Overfitting means that the function follows the data too closely, including data that might just be “noise.” This is especially harmful for prediction models as it leads to poor generalization of predictions on new data. This concept is also related to the so-called bias–variance trade-off [26]. As the flexibility of the spline increases, it better fits the data, reducing bias (i.e. reduces error due to underfitting). However, this flexibility also makes the spline more sensitive to fluctuations in the data, increasing variance (and the risk of overfitting). Conversely, reducing flexibility leads to lower variance (more of a straight line with less fluctuations) but increases bias as the straighter spline might not capture the underlying trend as well. Below, we go into more detail on how to approach this trade-off using a data driven method.

### Generalized additive models

Splines can be incorporated into various types of models; they can be used not only to deal with non-linearity in simple linear regression models, but also to deal with non-linearity in other types of models that rely on the assumption of a linear relationship (i.e. logistic regression). Many of these models are included under the umbrella of a broader class of models referred to as generalized linear models (GLMs). GLMs provide a framework to model outcome variables that do not follow the traditional normal distribution assumed by simple linear regression, such as the binary outcome in logistic regression, or a count outcome in Poisson regression [27]. It does so by using a so-called “link function” (e.g. the logit for logistic regression) which links the combination of independent variables in the model (i.e. “the linear predictor”) into a form that matches the outcome variable's distribution (the binomial distribution in the case of logistic regression). Irrespective of the type of outcome, GLMs assume a linear relationship between the (link function–transformed) outcome and the independent continuous variable.

While GLMs extend linear regression by allowing other types of outcomes, generalized additive models (GAMs) further extend GLMs by relaxing the linearity assumption [28, 29]. GAMs achieve this by replacing the linear form of GLMs with smooth functions of the independent variables. These smooth functions are combined additively to estimate the outcome. A main advantage of GAMs according to their creators is that they are “completely automatic, i.e. no ‘detective work’ is needed on the part of the statistician” [29]. As described above, regression splines require the user to manually define the number and placement of knots, which control the flexibility and smoothness of the fitted curve. GAMs, however, can “automate” this process using a penalty parameter [30]. This penalty parameter controls the bias–variance trade-off described above; higher penalties produce smoother curves (less variance) that may follow the underlying data less accurately (more bias), while lower penalties allow the curve to fit the data more tightly (less bias) with stronger fluctuations in the curve (more variance). High bias may lead to underfitting, whereas high variance may lead to overfitting. The size of the penalty parameter is typically based on the second derivative of the curve. While the first derivative represents the rate of change of the curve (i.e. the slope), the second derivative measures the rate of change of the slope itself (i.e. the slope of the slope, or curvature). A straight regression line, having a constant slope, would have a second derivative of zero [31]. GAMs use this second derivative to penalize the curve for excessive curvature, balancing model fit with smoothness (i.e. the bias–variance trade-off). The “automatic” element of GAMs, that eliminates the need for manual selection of knots, is the selection of the optimal value of the penalty parameter using a data-driven approach, often using cross-validation. This data-driven approach makes GAMs particularly useful when the underlying relationship is unknown [32]. Nonetheless, even with automation, GAMs are not foolproof, and it remains important to exercise caution when applying GAMs, as they can still be susceptible to overfitting. Generalized Additive Models for Location, Scale and Shape (GAMLSS) provide even more flexibility than GAMs as they further relax distributional assumptions such as homoscedasticity, and are recommended by the World Health Organization for the construction of growth curves [33, 34].

## EXAMPLE OF ANALYZING AND REPORTING A NON-LINEAR RELATIONSHIP

### Example description

In this example, we use data from the European QUALity Study on treatment in advanced CKD (EQUAL), an international observational cohort study on advanced CKD. In a previously published study, EQUAL explored non-linear trajectories of various clinical indicators in the years preceding death [35]. In this analysis, death was defined at a fixed time point (t = 0), and clinical indicator trajectories were examined up to 4 years prior to death (t = –4). In the main paper, as indicators evolved non-linearly over time, GAMs were applied to explore their trajectories. Below, we will apply the abovementioned methods to address non-linearity, focusing on the population average trajectory of serum albumin. While beyond the focus of this paper, when analyzing repeated measurements in patients over time, appropriate methods like linear mixed models are required [36]. This is because repeated measurements from the same patient are likely to be correlated, and failing to account for this correlation can lead to misleading results.

### Assessing the shape of the trajectory of serum albumin over time

**Figure 6:**
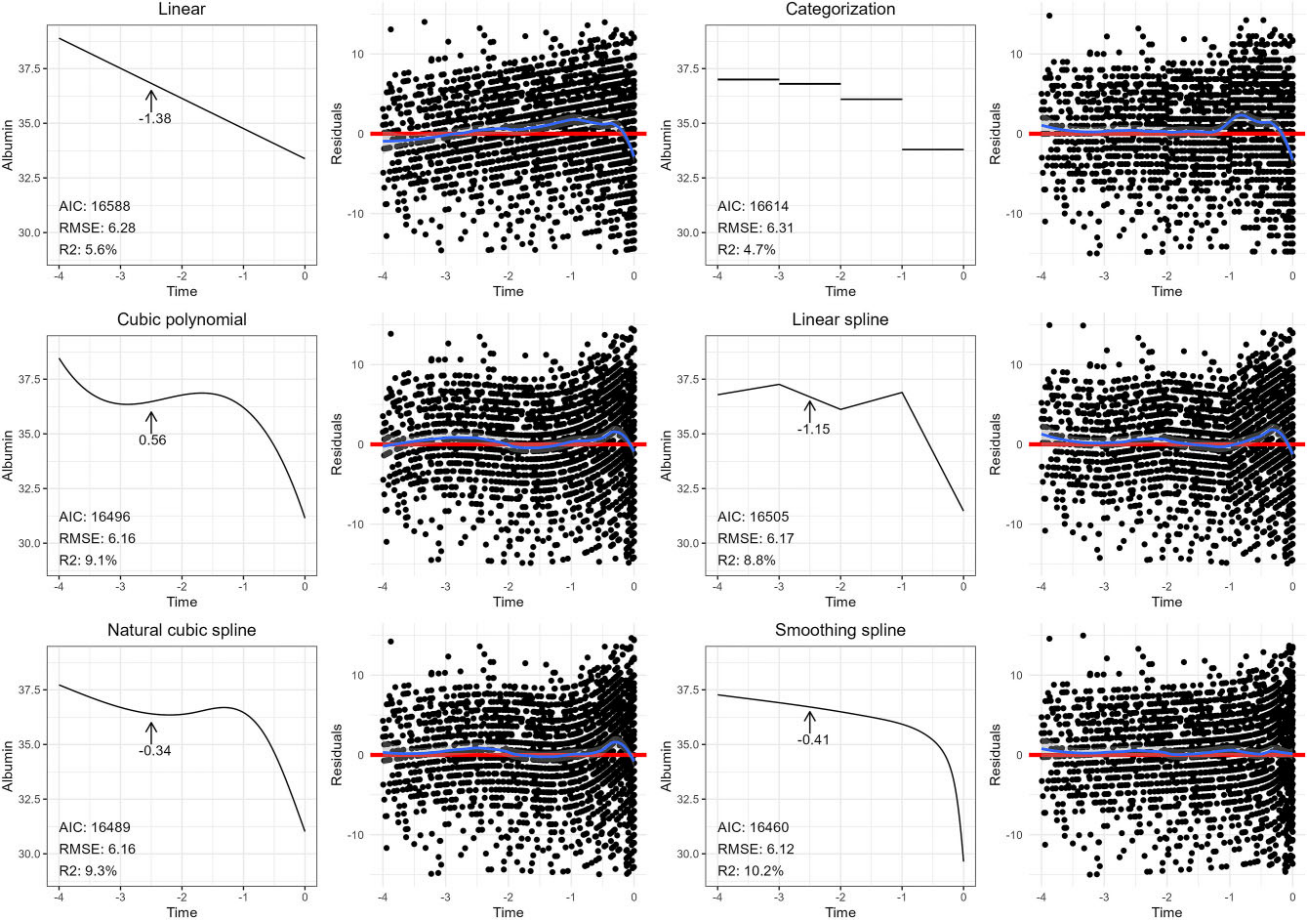
Population-average trajectories of serum albumin in the 4 years preceding death in advanced CKD patients, using various non-linear modeling methods, and as a function of time (blue line). We also included corresponding plots of residuals, with the blue line representing the smoothed average of the residuals as a function of time, and the red line representing the fitted curve. The RMSE (the square root of the average of the squared residuals), R-squared and the AIC are provided for each method. The slope (i.e. first derivative) is provided at –2.5 years preceding death for all methods except categorization (as the slope in the corresponding category is zero).

In this example, we assessed the effect of continuous time on the continuous outcome serum albumin. As a first step in the modeling process, we create a scatterplot of the raw data to visualize patterns, which will help guide our choice of modeling options. For the sake of the example presented in Fig. 6, the relationship was first modeled using linear regression, which suggests a poor fit: the residuals (moving average represented by the blue line) deviate from a random scatter around the regression line (red line), showing a curved pattern on both sides. This suggests that the linear regression model both over- and underestimates the true trajectory; from approximately –4 to –3 years, the model tends to overestimate serum albumin levels (as illustrated by the concentration of residuals under the curve), whereas from –1.5 to –0.25 years the model seems to underestimate the serum albumin levels. In the last 0.25 years of follow-up, the linear model clearly overestimates the true trajectory. This information will help us understand the shape of the non-linear relationship and aid in the decision on which methods to apply.

### How to select the best model for modeling the non-linear trajectory of serum albumin over time

As linear regression alone does not fit the trajectory of serum albumin over time, in Fig. 6 we explore several alternatives. (i) Linear regression using time categorized into four intervals to capture changes in trend, (ii) linear regression with time modeled as a cubic polynomial, (iii) linear regression with time modeled as a piecewise linear spline with four segments (knots placed at –3, –2 and –1 years), (iv) linear regression with time modeled as a natural cubic spline with four knots (placed at the 1st, 33rd, 66th and 99th percentiles) and (v) a GAM modeling time as a spline with automated smoothness selection.

The many options available to us raises the question on how to select the best model? Besides visual inspection of the fitted curve over the raw data, as well as the residual plots, there are several “goodness-of-fit” metrics available to help evaluate how well a model captures a relationship. The “root mean squared error” (RMSE) is the square root of the average of the squared residuals, and is a measure for the difference between the true values of the independent variable and the fitted values from the model, with lower values indicating a better model fit. Another commonly used metric to help in the selection of the best model is the Akaike Information Criterion (AIC), with lower values indicating a better fit. This metric helps find a balance between how well a model fits the data and the model's complexity. By including a penalty term for overly complex models (i.e. a model containing many covariates), the AIC helps reduce the risk of overfitting when used to select a model [37]. The R-squared statistic is another metric of model fit, and represents the proportion of the variance in the outcome that can be explained by the independent variables in the model, with higher values indicating a better fit [38]. The adjusted version of the R-square statistic also penalizes model complexity (represented by the number of covariates in the model) [39]. The RMSE, adjusted R-squared and the AIC are provided for each method in Fig. 6. Comparing these statistics among models indicates that the GAM may provide the best fit. Visual inspection of the residual plots leads to the same conclusion. If, however, two models were to have comparable performance, the principle of parsimony suggests opting for the simpler, least complex model.

### Reporting results for a non-linear relationship

While model fit metrics are useful, the choice of model also involves a trade-off between accuracy and the ease of communicating results. Simpler models, such as linear splines, offer easy interpretation, but at the cost of reduced accuracy. However, the results from “complex” spline models can be presented in an intuitive manner, and therefore there is little reason to sacrifice model accuracy for simplicity.

In a linear regression model, the effect of the independent variable, “time” in this example, is reflected by a single model coefficient (i.e. the change in serum albumin for a 1-unit increase in time), making it straightforward to interpret. Similarly, using a linear spline allows for a similar interpretation, as each segment within the spline has its own coefficient. Categorizing time into intervals also provides a straightforward way to report a non-linear relationship (despite its shortcomings), as it allows simply communicating the mean serum albumin within each time period.

Unlike linear relationships, which assume a constant effect of the independent variable, models containing splines or polynomials have effects that change over the range of the variable. In our non-linear example, we cannot summarize our findings with a single regression coefficient as the “effect” of time on serum albumin varies throughout the follow-up period, making interpretation less straightforward. Several methods exist to report results in this situation. The easiest is to simply present the curve visually in a plot, showing how the trajectory of serum albumin evolves over time. However, some form of quantitative assessment is often desired to complement the plots. One approach is to report the slope (i.e. regression coefficient) at multiple time points of interest. The slope can be calculated by determining the difference in serum albumin levels between specific time points. For example, estimating the change in serum albumin between –3 and –2 years would reveal the effect (i.e. slope) of time on serum albumin during that specific interval. In other words, the calculated slope—or regression coefficient—reflects the effect for a 1-unit increase in time on serum albumin, specifically within that time interval. The calculated slope for the interval between –1 and 0 years would likely show a stronger effect of time, as visualized by the steeper slope in that part of the curve. Changes in slope indicate periods of more rapid change (or stabilization) in serum albumin. Of note, this method provides the effect at the midpoint between the time values (i.e. the average slope over the time interval). Another more accurate approach to understand the fluctuating effect of time is to calculate the first derivative of the trajectory. This provides the instantaneous rate of change (i.e. slope) at any point on the curve, and in our example, reveals how quickly albumin levels are rising or falling at different points during follow-up. As an example, Fig. 6 provides the slope (i.e. first derivative) at –2.5 years preceding death for all methods except categorization (as the slope in the corresponding category is zero), demonstrating how different the results can be depending on the method used.

## EXAMPLE OF ANALYZING AND REPORTING RESULTS FOR A NON-LINEAR RELATIONSHIP USING RELATIVE RISKS

In nephrology research, outcomes are often binary (yes/no) or time-to-event (e.g. patient survival). For binary outcomes, logistic regression is typically used, and for time-to-event outcomes, the Cox proportional hazards model is often preferred. Both models assume a linear relationship between the independent continuous variable and the log odds (for logistic regression) or the log hazard (for Cox regression). Results are often reported as relative risks; odds ratios for logistic regression and hazard ratios (HRs) for Cox models. Both represent the relative change in the outcome for every unit increase in the continuous independent variable.

**Figure 7:**
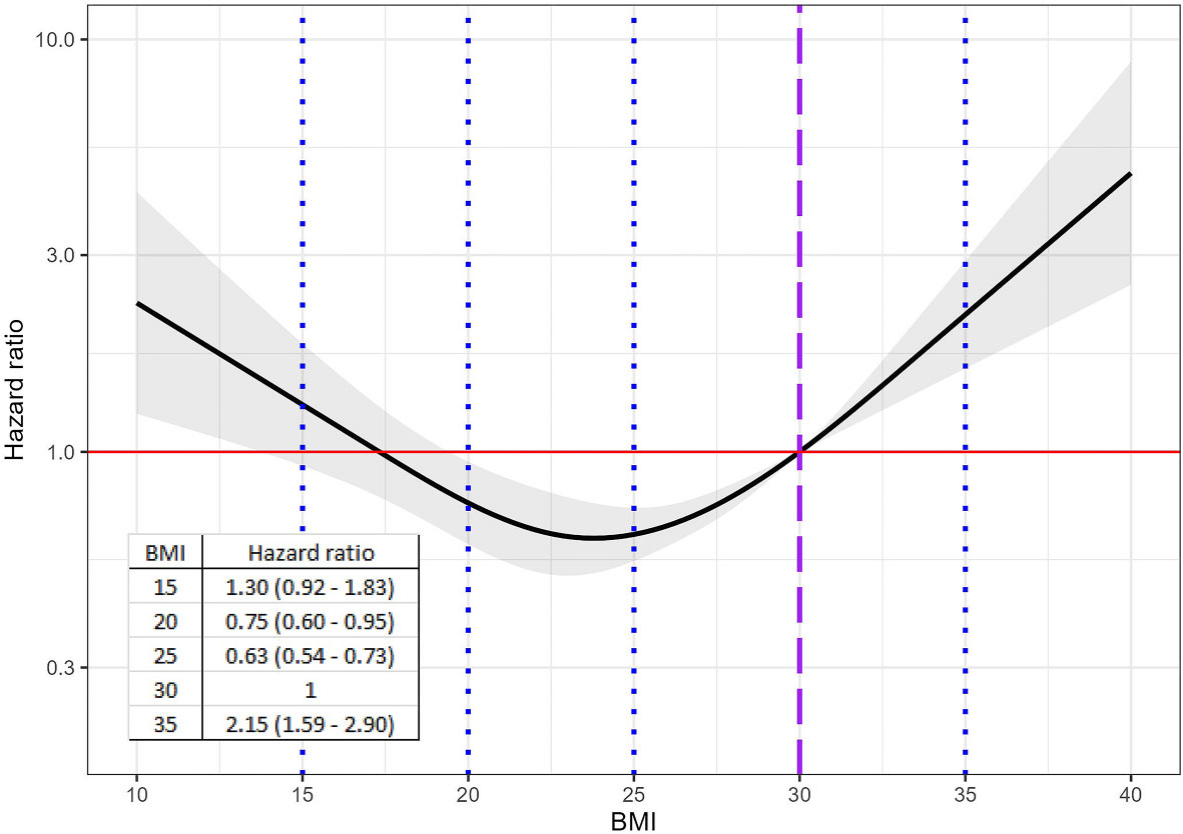
A natural cubic spline is used to model the non-linear relationship between BMI and mortality risk using a Cox regression model. A BMI (kg/m^2^) of 30 is used as the reference category for calculating HRs (purple dashed line). HRs are shown for BMI values of 15, 20, 25 and 35 (blue dotted lines). HRs above 1 (red line) indicate a higher risk of death relative to a BMI of 30. Conversely, HRs below 1 indicate a lower risk.

In this example, we use simulated data to explore the non-linear relationship (using restricted cubic splines) between a continuous independent variable (BMI) and a time-to-event outcome (mortality risk) in a Cox regression model (but the same approach can be taken for logistic regression presenting odds ratios). After having identified a non-linear relationship using splines exploratory to visualize the relationship between BMI and the relative risk of death, it was determined by comparing multiple models using goodness-of-fit metrics (e.g. AIC), that a natural cubic spline with four knots (placed at the 1st, 33rd, 66th and 99th percentiles) provided the best fit. Results are presented both graphically in Fig. 7, and quantitively by comparing selected values of BMI to a chosen reference value of BMI. In this case, we set the reference value to a BMI of 30 kg/m^2^, and present the HRs for the BMI values 15, 20, 25 and 35 kg/m^2^. For example, the HR for a BMI of 20 was 0.75. This means that individuals with a BMI of 20 were 25% less likely to die during the study period compared with those with a BMI of 30. The plot can be reproduced using the R code provided in the Supplementary data.

## CONCLUSION

“Essentially all models are wrong, but some are useful.” George Box reminds us that although statistical models are unable to perfectly capture the complexities of the real world, they still offer a convenient approximation, which can be useful to clinical practice [40]. Despite the characteristic of all statistical models inherently being “wrong,” in epidemiological research, we strive to produce models that are as truthful to reality as possible. As nonlinear relationships arguably occur more often than linear ones, appropriate methods are required to ensure that the modeled relationship follows the data at hand, and that the shape of the relationship is accurately represented by the model. Here we provide the strengths and limitations of the various tools capable of modeling non-linear relationships, including transformations, polynomials, splines and GAMs, whilst demonstrating how the reporting of results using these techniques can be surprisingly straightforward, despite their perceived complexity.

## Supplementary Material

gfae187_Supplemental_File

## Data Availability

Figures 2, 3 and 7 can be reproduced using the R code provided in the Supplementary data.
