# Supplementary material for: Non-linear relationships in clinical research
Source: Nephrol Dial Transplant. 2024 Aug 21;40(2):244–54. doi: 10.1093/ndt/gfae187 (PMC11852331; doi:10.1093/ndt/gfae187)
Supplement: gfae187_Supplemental_File [file gfae187_Supplemental_File.docx]

**Supplementary materials**

R code to reproduce Figure 2

# Importing libraries

library(qqplotr)

library(dplyr)

library(gridExtra)

library(ggplot2)

library(splines)

library(mgcv)

set.seed(124) # Set seed for reproducibility

# Simulate data

x <- seq(1, 200, 1)

y <- 2 + 0.4 * x + rnorm(200, sd = 8) # Variance increases with x

# Linear regression model - residuals and fitted values

model <- lm(y ~ x)

residuals <- residuals(model)

fitted_values <- fitted(model)

# Scatterplot and regression line

A1<-ggplot(data = data.frame(x, y), aes(x = x, y = y)) +

geom_point(color = "dodgerblue3") +

geom_smooth(method = "lm", se = FALSE, color = "red", linewidth=1)+

labs(x = "X", y = "Y") +

theme_bw()+

geom_text(aes(x=-Inf,y=Inf,hjust=-1,vjust=2,label="A1",fontface="bold"))

A1

# Residual plot

A2<-ggplot(data = data.frame(fitted_values, residuals), aes(x = fitted_values, y = residuals)) +

geom_point(color = "dodgerblue3") +

geom_abline(slope = 0, intercept=0, col = "red",linewidth=1)+

labs(x = "Fitted Values", y = "Residuals") +

theme_bw()+

geom_text(aes(x=-Inf,y=Inf,hjust=-1,vjust=2,label="A2",fontface="bold"))

A2

# Normal probability plot

slope <- sd(residuals)

int <- mean(residuals)

d <- data.frame(resids = residuals)

A3 <- ggplot(d, aes(sample = resids)) + stat_qq(color = "dodgerblue3") +

geom_abline(slope = slope, intercept = int, col = "red",linewidth=1)+

labs(x = "Theoretical Quantiles", y = "Residuals") +

theme_bw()+

geom_text(aes(x=-Inf,y=Inf,hjust=-1,vjust=2,label="A3",fontface="bold"))

A3

#Histograms of residuals

A4<-ggplot(d, aes(x = resids)) +

geom_histogram(fill = "grey", color = "black") + # Customize binwidth, colors if desired

labs(x = "Residuals",y = "Count") +

theme_bw()+

geom_text(aes(x=-Inf,y=Inf,hjust=-1,vjust=2,label="A4",fontface="bold"))

A4

#Non-linear example

set.seed(123) # Set seed for reproducibility

# Simulate data

n <- 250

X <- rnorm(n, mean = 0, sd = 1)

Y <- X^2 + 2*X + 3

epsilon <- rgamma(n, shape = 2, scale = 1) # Right-skewed gamma distribution

Y <- Y + epsilon

y <- Y

x <- X

model <- lm(y ~ x)

residuals <- residuals(model)

fitted_values <- fitted(model)

hist(y)

annotations <- data.frame(

xpos = c(-Inf),

ypos = c(Inf),

annotateText = c("B1"),

hjustvar = c(-1) ,

vjustvar = c(2)) #<- adjust

# Scatterplot

B1<-ggplot(data = data.frame(x, y), aes(x = x, y = y)) +

geom_point(color = "dodgerblue3") +

geom_smooth(method = "lm", se = FALSE, color = "red", linewidth=1)+

labs(x = "X", y = "Y") +

theme_bw()+

geom_text(aes(x=-Inf,y=Inf,hjust=-1,vjust=2,label="B1",fontface="bold"))

B1

# Residual plot

B2<-ggplot(data = data.frame(fitted_values, residuals), aes(x = fitted_values, y = residuals)) +

geom_point(color = "dodgerblue3") +

geom_abline(slope = 0, intercept=0, col = "red",linewidth=1)+

labs(x = "Fitted Values", y = "Residuals") +

theme_bw()+

geom_text(aes(x=-Inf,y=Inf,hjust=-1,vjust=2,label="B2",fontface="bold"))

B2

# Normal probability plot

slope <- sd(residuals)

int <- mean(residuals)

d <- data.frame(resids = residuals)

B3 <- ggplot(d, aes(sample = resids)) + stat_qq(color = "dodgerblue3") +

geom_abline(slope = slope, intercept = int, col = "red",linewidth=1)+

labs(x = "Theoretical Quantiles", y = "Residuals") +

theme_bw()+

geom_text(aes(x=-Inf,y=Inf,hjust=-1,vjust=2,label="B3",fontface="bold"))

B3

#Histograms of residuals

B4<-ggplot(d, aes(x = resids)) +

geom_histogram(fill = "grey", color = "black") + # Customize binwidth, colors if desired

labs(x = "Residuals",y = "Count") +

theme_bw()+

geom_text(aes(x=-Inf,y=Inf,hjust=-1,vjust=2,label="B4",fontface="bold"))

B4

plot<-grid.arrange(A1, B1, A2, B2, A3, B3, A4, B4, ncol=2, nrow=4, widths=c(1,1), heights=c(1,1,1,1)

R code to reproduce Figure 3

# Simulate data

set.seed(123) # For reproducibility

n <- 200

x <- runif(n, min = 0, max = 10)

y <- (3*x^4 - 3*x + rnorm(n, sd = 1500) )/1000

data <- data.frame(x, y)

# Models

data$x_cat <- cut(data$x, breaks = c(0, 3.33, 6.66, 10),

labels = c("low", "medium", "high"))

model_continuous <- lm(y ~ x, data = data)

model_categorical <- lm(y ~ x_cat, data = data)

model_ns <- lm(y ~ ns(x,3), data = data)

model_linear_spl <- lm(y ~ bs(x, knots = c(2.5, 7.5), degree=1), data = data)

summary(model_categorical)

fig2<-ggplot(data, aes(x = x, y = y)) +

geom_point() +

geom_line(aes(y = predict(model_continuous), color = "blue"), color = "blue",size = 1) +

geom_segment(aes(x = 0, xend = 3.33, y = 0.2062, yend = 0.2062, color = "red"), size = 1.5, linetype = "dashed")+

geom_segment(aes(x = 3.33, xend = 6.66, y = 2.1908, yend = 2.1908, color = "red"), size = 1.5, linetype = "dashed")+

geom_segment(aes(x = 6.66, xend = 10, y = 15.4996, yend = 15.4996, color = "red"), size = 1.5, linetype = "dashed")+

geom_line(aes(y = predict(model_ns)), color = "purple", size=1) +

geom_line(aes(y = predict(model_linear_spl)), color = "green", size=1) +

labs(x = "Independent Variable", y = "Outcome") +

theme_bw()

fig2<-fig2 + theme(legend.position = "none")

fig2

R code to reproduce Figure 7

# Libraries

library(survival)

library(rms)

library(ggplot2)

# Simulate data

set.seed(123) # Set seed for reproducibility

# Sample size

n <- 650

# True effect (U-shaped)

beta1 <- 1 # Linear effect

beta2 <- -0.08 # Quadratic effect

# Generate continuous covariate (x) with U-shaped distribution

x <- rnorm(n, mean = 5, sd = 1.7)

x2 <- x*3+x^2+-2*x^-0.5

# Baseline hazard (assumed to be unspecified)

u <- runif(n)

# Generate event times (t)

t <- -log(u) * exp(beta1*x + beta2*x2)

# Generate censoring indicator (event = 1, censored = 0)

# Adjust censoring rate to achieve some censored data

censored_prop <- 0.2

event <- rbinom(n, 1, 1 - censored_prop)

# Combine data

data <- data.frame(t = t, event = event, x = x)

dd <- datadist(data)

options(datadist="dd")

# RMS - Cox model

rms.spline<- cph(Surv(t, event) ~ rcs(x,4), data=data)

AIC(rms.spline)

# Set reference value

dd$limits["Adjust to","x"] <- 6.6666

rms.spline <- update(rms.spline)

# Output predictions

pred<-rms::Predict(rms.spline, x=seq(0,10, by=0.1), ref.zero=T, fun=exp)

# Scale x to a reasonable range of BMI

pred$x<-(pred$x*3)+10

pred<-as.data.frame(pred)

# Plot

p<-ggplot(pred, aes(x=x, y=yhat)) +

geom_line(linewidth =1) +

geom_ribbon(aes(ymin = lower, ymax = upper), alpha = 0.1, linetype=2)+

labs(x="BMI",y="Hazard ratio")+

theme_bw()+

geom_vline(xintercept = 30, linetype = 5, colour = "purple",linewidth =1)+

geom_vline(xintercept = 25, linetype = 3, colour = "blue", linewidth =1)+

geom_vline(xintercept = 35, linetype = 3, colour = "blue", linewidth =1)+

geom_vline(xintercept = 15, linetype = 3, colour = "blue", linewidth =1)+

geom_vline(xintercept = 20, linetype = 3, colour = "blue", linewidth =1)+

geom_hline(yintercept = 1, colour = "red") +

scale_y_log10(limits = c(0.2, 10))+

scale_x_continuous(breaks = round(seq(min(pred$x), max(pred$x), by = 5),1))

p
